# Validation of the Surgical Fear Questionnaire in Adult Patients Waiting for Elective Surgery

**DOI:** 10.1371/journal.pone.0100225

**Published:** 2014-06-24

**Authors:** Maurice Theunissen, Madelon L. Peters, Erik G. W. Schouten, Audrey A. A. Fiddelers, Mark G. A. Willemsen, Patrícia R. Pinto, Hans-Fritz Gramke, Marco A. E. Marcus

**Affiliations:** 1 Department of Anesthesiology and Pain Management, Maastricht University Medical Center+, Maastricht, the Netherlands; 2 Department of Clinical Psychological Science, Maastricht University, Maastricht, the Netherlands; 3 Life and Health Sciences Research Institute (ICVS), School of Health Sciences, University of Minho, Braga, Portugal; ICVS/3B's – PT Government Associate Laboratory, Braga/Guimarães, Portugal; 4 Department of Anesthesia/ICU, Pain and Palliative Care, Hamad Medical Corporation, Doha, Qatar; Research and Development Corporation, United States of America

## Abstract

**Objectives:**

Because existing instruments for assessing surgical fear seem either too general or too limited, the Surgical Fear Questionnaire (SFQ) was developed. The aim of this study is to assess the validity and reliability of the SFQ.

**Methods:**

Based on existing literature and expert consultation the ten-item SFQ was composed. Data on the SFQ were obtained from 5 prospective studies (N = 3233) in inpatient or day surgery patients. These data were used for exploratory factor analysis (EFA), confirmatory factor analysis (CFA), reliability analysis and validity analysis.

**Results:**

EFA in Study 1 and 2 revealed a two-factor structure with one factor associated with fear of the short-term consequences of surgery (SFQ-s, item 1–4) and the other factor with fear of the long-term consequences of surgery (SFQ-l, item 5–10). However, in both studies two items of the SFQ-l had low factor loadings. Therefore in Study 3 and 4 the 2-factor structure was tested and confirmed by CFA in an eight-item version of the SFQ. Across all studies significant correlations of the SFQ with pain catastrophizing, state anxiety, and preoperative pain intensity indicated good convergent validity. Internal consistency (Cronbach's alpha) was between 0.765–0.920 (SFQ-total), 0.766–0.877 (SFQ-s), and 0.628–0.899 (SFQ-l). The SFQ proved to be sensitive to detect differences based on age, sex, education level, employment status and preoperative pain intensity.

**Discussion:**

The SFQ is a valid and reliable eight-item index of surgical fear consisting of two subscales: fear of the short-term consequences of surgery and fear of the long-term consequences.

## Introduction

Preoperative or surgical fear is a well recognizable emotional state for many patients waiting for surgery and is a risk factor for major personal and socio-economic burden. Various studies have found that surgical fear is associated with impaired psychosocial and physical recovery, such as increased levels of acute and chronic postoperative pain [1]–[3]. Therefore, preoperative assessment of surgical fear could provide essential information for improving perioperative care and could be a first step towards targeted intervention.

Objects of surgical fear can be heterogeneous. Previous studies have listed more than 20 objects of fear, varying from fear of the surgical procedure itself to fear of the anaesthesia, having to undergo blood transfusions, being stung with needles, losing dignity or even dying [4]–[6]. Some factors that may influence the reported prevalence of surgical fear are type or impact of planned surgery, time span until surgery, previous experience with surgery, provision of preoperative information about surgical procedure, age and sex [3], [5], [7]–[9]. Also, the instrument used for assessment of fear may influence the reported prevalence.

Only few instruments are available for assessment of surgical fear and most of these are disease specific, such as the Bypass Grafting Fear Scale (BGFS) [10] and the Surgery Stress Scale (for knee surgery) [11]. Therefore, in many studies, nonspecific instruments have been used such as the Hospital Anxiety and Depression Scale (HADS) [12], State-Trait Anxiety Inventory (STAI) [13], or a Visual Analogue Scale (VAS) assessing anxiety. One generic instrument has been developed for preoperative assessment of surgical fear, the six-item Amsterdam Preoperative Anxiety and Information Scale (APAIS) [14], [15]. However, this instrument is relatively limited in scope; it includes two items on fear of the anaesthetic procedure and two items on fear of the surgical procedure. The remaining two items asses the need for information rather than fear.

Because existing instruments for assessing surgical fear are either limited in scope, or too general, or too specific and not broadly generalizable to other surgical populations, we developed the Surgical Fear Questionnaire (SFQ). The SFQ has already been used in several studies [16]–[22] but formal assessment of its validity and reliability is still lacking. This paper describes the development and psychometric assessment of the SFQ. Similar to the BGFS [10], the SFQ aims to be comprehensive enough to cover the most important targets of fear and at the same time concise enough for general use in clinical practice and research. We present data on the construct, content, convergent, and predictive validity as well as the internal consistency of the SFQ. Data from five different studies in which the SFQ was administered to patients one day to one week prior to undergoing inpatient or day surgery are used. Because patients from different clinical populations and different countries are included, this also allows us to test the stability of the SFQ and its factor structure across different subgroups.

## Materials and Methods

### Ethics statement

Study 1, 3, 4, and 5 were approved by the Medical Ethics Committee of Maastricht University Medical Center+, Maastricht, the Netherlands. For Study 2 approval was given by the Medical Ethics Committee of the Centro Hospital do Alto Ave, Guimarães, Portugal. All patients gave written informed consent.

### Scale development

The SFQ was developed to create a tailor made instrument for the assessment of self-reported surgical fear, suitable for general use among all types of adult surgery patients, and covering a broad range of short-term and long-term surgery-related fears. The composition and phrasing of the SFQ was based on items selected from existing questionnaires [4], [6], [10], [14], [23] and expert consultation. The selection of the initial 10 items took place after a consensus meeting of experts in the field of psychology, anaesthesiology, methodology, or epidemiology. All items are scored on an eleven point numeric rating scale (NRS) ranging from 0 (not at all afraid) to 10 (very afraid). This results in a total score of 0 to 100. Selected items are: afraid of operation, anaesthesia, postoperative pain, side effects, health deterioration, failed operation, hospital stay, (worried) about family members, incomplete recovery, long duration of rehabilitation.

### Procedure

To establish the factor structure of the SFQ, data of four different studies were used, see table 1. A two stage approach was employed. Exploratory factor analysis (EFA) was performed on the data of the first two studies, followed by confirmatory factor analysis (CFA) on the data of study 3 and 4. EFA is used to explore the underlying factor structure of a set of items without an a priori hypothesis about the number and structure of factors to be identified. CFA is a hypothesis testing technique used to confirm the solution of the EFA in a different sample.

**Table 1:** Sociodemographic and surgery characteristics of Study 1–5.

	Study 1	Study 2	Study 3	Study 4	Study 5
	N = 1490	N = 201	N = 192	N = 1275	N = 75
Country					
	NL	P	NL	NL	NL
Surgery					
	Mixed inpatient	Hysterectomy	Hysterectomy	Mixed	Mixed
				day surgery	day surgery
Age					
	55.6±15.5	51.2±9.4	46.2±7.8	51.9±14.7	52.8±15.3
Sex					
Male	702	-	-	722	31
Female	788	201	192	553	44
Education					
Low	392	188	33	396	20
Intermediate & high	788	12	158	864	52
Missing	310	1	1	15	3
Employment					
Occupation	484	99	129	688	34
No occupation	684	102	60	586	37
Missing	322	0	3	1	4
ASA					
I/II	1222	184	180	1196	69
III/IV	268	14	3	53	6
Missing	0	3	9	26	0
Malignancy					
Yes	239	0	0	107	7
No	1251	201	192	1168	68
Preoperative pain					
	3 (0–21)	40 (20–50)	50 (30–60)	20 (0–50)	30 (0–60)
Expected pain					
No/mild	760	48	47	590	28
Moderate/high	679	52	142	651	47
Missing/don't know	51	101	3	34	0

N numbers baseline population; mean ± standard deviation, median (interquartile range).

Country: NL the Netherlands, P Portugal.

- Not applicable. Preoperative pain: VAS/NRS 0–100. Expected pain VAS/NRS 0–100 or Likert scale (Study 2): no/mild pain VAS/NRS <40, moderate/high pain VAS/NRS 40–100. ASA: American Society of Anesthesiologists.

An initial EFA was performed on the SFQ data obtained from a prospective observational cohort study examining predictors of acute and chronic postoperative pain [16], [17]. The sample consisted of 1490 Dutch inpatients scheduled to undergo surgery at one of the following departments: general surgery, plastic surgery, orthopedics, ophthalmology, gynaecology, ear-nose-throat, maxillofacial surgery, urology, neurosurgery, or thoracic surgery. Table 1 presents the primary sample characteristics (Study 1). Patients completed the SFQ in the hospital one day before surgery.

To examine the robustness of the factor solution, the EFA was repeated in a second independent sample consisting of 201 women. Data were obtained from a prospective cohort study on predictors of acute and chronic pain after elective hysterectomy carried out in Portugal. The sample characteristics are described in table 1 (Study 2) [18]. In a face to face interview with a trained psychologist the SFQ was completed on the day before surgery in the hospital. For the translation of the SFQ into Portuguese a three stage procedure was performed. The first step was the forward translation of the English version into Portuguese. This was done by a bilingual person, a native speaker of the target language (Portuguese). The second step was a separate back translation. This was performed by a bilingual translator who is a native speaker of the source language (English). The translations coincided. In step three a pilot with the Portuguese version of the SFQ was performed in a sample of 46 women undergoing hysterectomy. Before surgery the SFQ was applied and participants were asked to reflect on the comprehensibility of the scale and asked for additional suggestions. The women agreed with the Portuguese translated version and showed no doubts about the items. After this the Portuguese version of the SFQ was considered ready for use.

Meanwhile a new (Brazilian) Portuguese translation was made from the original Dutch version of the scale (A.C. Mesquita, University of São Paulo at Ribeirão Preto College of Nursing). Back translation to Dutch of this version showed it to be 100% identical to the original version. This new Portuguese translation was compared to the version used in study 2. There were only minor differences in wording which are mostly due to differences between Brazilian and European Portuguese.

On the basis of the results obtained in Study 1 and 2 two items were deleted from the SFQ and a two-factor structure was proposed for the new eight-item SFQ yielding a range of 0–80 (see below). Confirmatory factor analysis (CFA) was used to test the fit of this two-factor model against a one-factor model in a new sample of hysterectomy patients [24]. Data were obtained from the first 192 included patients of an ongoing prospective multicenter study in the Netherlands on predictors of postoperative recovery after hysterectomy. Sample characteristics are presented in table 1 (Study 3). These patients completed the eight-item version of the SFQ at home in the week before surgery.

In the last step we tested the invariance of the factor structure in a mixed male – female sample of patients undergoing various surgical procedures. Data were obtained from a prospective cohort study on the prevalence of postoperative pain in adult patients after elective day surgery performed in the Netherlands [25]. The most frequently performed types of surgery in this study were, among other, general surgery, orthopaedic surgery, ear nose throat surgery, plastic surgery, and gynaecologic surgery. Sample characteristics are presented in table 1 (Study 4). A total of 1275 patients completed the eight-item version of the SFQ at home in the week before surgery.

Convergent validity was tested by comparing the scores on the SFQ with scores on questionnaires assessing general anxiety, or negative cognitions about pain before the operation. All four studies that provided data for the psychometric evaluation of the SFQ included a measure of pain catastrophizing, either the Pain Catastrophizing Scale (PCS) or the catastrophizing subscale of the Coping Strategies Questionnaire-revised (CSQ-R) [26], [27]. Both scales measure negative cognitions and worrying about pain. The full thirteen-item PCS was included in Study 1 and 3, a six–item abbreviated version in Study 4 and the catastrophizing subscale of CSQ-R was used in Study 2. Additionally, all studies included a pre-operative assessment of expected pain. Only Study 2 also included a measure of pre-operative general anxiety, namely the anxiety subscale of the HADS. The HADS is a widely used and well validated instrument, developed for assessing self-reported anxiety and depression [28]. For the PCS [29], CSQ-R [30] and HADS [31] validity of the Portuguese and Dutch versions has been established.

Because one of the most frequently used instruments for measuring pre-operative (general) anxiety is the STAI [2], and this instrument was not included in any of the previous studies with the SFQ, we performed an additional study (Study 5) to assess convergent validity of the SFQ with the STAI. The Dutch version of the STAI was has been shown to be valid [32]. Both the state and trait anxiety subscales were included. In addition, patients filled out the PCS and the numerical rating scale to assess expected pain intensity. Study 5 included 75 adult patients scheduled for elective day surgery. Inclusion criteria and types of operation were similar as in study 4. All questionnaires were completed at home in the week before surgery.

Besides construct and convergent validity, also the internal consistency of the SFQ was assessed. Therefore, in all studies Cronbach's alpha was calculated.

The next step in the validation procedure was the assessment of the sensitivity to detect differences in fear between subgroups based on age, sex, employment status, ASA (American Society of Anesthesiologists) classification, surgery because of malignancy (yes/no), preoperative pain status (no/mild or VAS/NRS <40, moderate/high or VAS/NRS 40–100) [33], and education (lower compared to intermediate/higher education). Lower education was defined as no education, primary education, lower vocational education, or ≤9 years education (Study 2). Intermediate education was defined as secondary education, intermediate vocational education, or 10–12 years of education (Study 2). Higher education was defined as higher vocational education, university, or graduation (Study 2). Finally, predictive validity was assessed on data of Study 1 and 4. Predictor variables were the SFQ and its subscales, dichotomized by median split [16]. Outcome measures were acute postsurgical pain on postoperative day 4 and chronic postsurgical pain, after 6 months in Study 1 and after one year in Study 4. Another outcome measure for predictive validity was self-perceived recovery, assessed by the global surgical recovery index (GSR, range 0–100%, values of 80–100% were considered as good recovery) [16], [34].

### Statistical analysis

Parametric data were described using mean ± standard deviation, non-parametric data with median and interquartile range (IQR) and minimum-maximum values. EFA (principal component analyses) was performed using oblique factor rotation (oblimin). Factor extraction was based on evaluation of the scree plot and the Kaiser's criterion (factors with eigenvalues >1 were retained). Item selection was based on evaluation of factor loadings (cut-off value >0.40). The factor loadings can be thought of as the Pearson correlation between a factor and a variable. Item selection was further confirmed by reliability analysis (evaluation of Cronbach's alpha, values ≥0.7 are considered fair and ≥0.8 good). For CFA improvement of goodness of fit was assessed by Minimum Fit Function chi square. Other test criteria were the Root Mean Square Error of Approximation (RMSEA), Standardized Root Mean Square Residual (SRMR), Non-Normed Fit Index (NNFI), and the Comparative Fit Index (CFI). The RMSEA and SRMR reflect the deviation of the factor solution from the data (the lower the better, with a minimum of 0) and the NNFI and CFI reflect the deviation of the factor solution with the independence model (the higher the better, with a maximum around 1). Values indicating a good fit are for RMSEA ≤0.06, SRMR ≤0.09, NNFI and CFI ≥0.95 [35]. Convergent validity was assessed using the Pearson correlation coefficient. Sensitivity analysis was performed with the Mann Whitney U-test. For assessing predictive validity of the SFQ, odds ratios (OR) were generated by bivariate logistic regression analyses. For the descriptive statistics, EFA, reliability analysis, validity analysis, and sensitivity analysis the Statistical Package for the Social Sciences was used (SPSS version 18, Chicago, Illinois, USA). The CFA was performed with Lisrel 8.20 (Jöreskog & Sörbom, Scientific Software International, Chicago, Illinois, USA). A *p*-value <0.05 was considered statistically significant for all analyses except for the convergent and predictive validity analyses. To adjust for multiple testing a Bonferroni correction of 0.05∶3 was applied resulting in a *p*-value <0.017 considered statistically significant for all Pearson correlation coefficients and logistic regression analyses.

## Results

### Study 1. Exploratory factor analysis: initial results

The original 10-item version of the SFQ was completed by 1490 patients. A median score of 23 was obtained (IQR 11–38). In table 2 median and minimum-maximum scores are presented. The scores of all items comprised the whole range from 0–10 indicating an appropriate item scaling, although some floor effect cannot be excluded since the distribution is skewed to the right.

**Table 2:** SFQ scores of Study 1–5.

	Study 1	Study 2	Study 2	Study 3	Study 4	Study 5
	10 items	10 items	8 items	8 items	8 items	8 items
SFQ	23 (0–98)	20 (0–82)	13 (0–62)	22.9 (0–77)	22 (0–80)	25 (0–66)
SFQ-s	12 (0–40)	9 (0–36)	9 (0–36)	14 (0–40)	14 (0–40)	14 (0–38)
SFQ-l	9.5 (0–60)	10 (0–48)	3 (0–35)	7 (0–38)	8 (0–40)	9 (0–32)

Median (minimum-maximum).

SFQ-s: Surgical Fear Questionnaire short-time consequences (item 1–4), SFQ-l: SFQ long-term consequences (10-item version: item 5–10; 8-item version: item 5, 6, 9,10).

The EFA identified two factors together explaining 60.2% of the total variance. All items loaded adequately (defined as >0.40) on one of the two factors (see table 3). Inspection of the items indicated that the items of one of the factors referred to more proximal fears (item 1–4; e.g. fear of pain, fear of anaesthesia) while the items in the other factor referred to more distal fears (item 5–10; e.g. fear of incomplete recovery, fear of long rehabilitation). These factors were labelled “fear of the short-term consequences of surgery” (SFQ-s) and “fear of the long-term consequences of surgery” (SFQ-l) respectively. Cronbach's alpha of the SFQ-s was 0.83, of SFQ-l 0.82 and of the total scale 0.87. The intercorrelation between SFQ-I en SFQ-s was 0.57, *p*<0.01.

**Table 3:** Exploratory factor analysis.

		Study 1		Study 2	
		SFQ-s	SFQ-l	SFQ-s	SFQ-l
	Eigenvalue	1.211	4.807	3.588	1.440
1	Operation	0.845	0.035	0.889	0.091
2	Anaesthesia	0.907	−0.123	0.756	0.045
3	Pain	0.657	0.200	0.695	−0.073
4	Side effects	0.740	0.054	0.719	0.034
5	Health deterioration	0.040	0.768	0.066	−0.728
6	Failed operation	0.013	0.776	−0.068	−0.761
7	Hospital stay	0.256	0.434	0.393	−0.125
8	Family	0.156	0.464	0.316	−0.008
9	Incomplete recovery	−0.114	0.931	0.094	−0.805
10	Long rehabilitation	−0.057	0.834	−0.028	−0.770

Eigenvalues and factor loadings.

This initial EFA thus indicated a two-factor model for the SFQ comprising all ten items. To examine the robustness of this factor structure and the generalizability to a different population, the EFA was repeated using data from a Portuguese study on women undergoing hysterectomy.

### Study 2. Exploratory factor analysis: confirmation in an independent sample

The 10-item SFQ was completed by 201 patients. Compared to our previous sample, patients in this sample scored somewhat lower on most items with a median score of 20 (IQR 10–32), see also table 2. All ten items yielded scores ranging the full scale from 0–10. The distribution was skewed to the right.

EFA again revealed a two-factor structure similar to Study 1, explaining 50.3% of the variance. One factor contained items related to fear of short-term consequences of surgery (item 1–4) and one factor contained items related to long-term consequences of surgery (item 5, 6, 9, 10). However, two items (item 7: “I am afraid of staying in the hospital” and item 8 “I worry about my family”) did not load above the cut-off of >0.40 on either of the two factors (table 3). These were also the two items that had the lowest factor loading in the previous sample, with loadings well below the other items on the same factor. Moreover, Cronbach's alpha on the SFQ-l subscale indicated only moderate internal consistency (0.63). Deleting these two insufficiently loading items increased the Cronbach's alpha of the SFQ-I subscale to 0.77. Cronbach's alpha of the total scale increased from 0.77 to 0.80; Cronbach's alpha for SFQ-s was 0.77. Intercorrelation between the SFQ-s and SFQ-l subscale was 0.41, *p*<0.01. A post hoc reliability analysis on the SFQ eight-item total scale and four-item SFQ-l subscale of Study 1 revealed a Cronbach's alpha of 0.87 on the total scale (unchanged) and of 0.84 on the SFQ-l.

Based on the factor loadings and internal consistency, the SFQ can best be used as an eight-item scale with two subscales, each consisting of four items. In the next step, we performed a confirmatory factor analysis on the eight-item SFQ in a new sample of women undergoing hysterectomy. We compared the two-factor model with a one-factor model. It may be argued that a one-factor model is equally suitable and more parsimonious for the data because of the high internal consistency of the total scale and the moderate but significant intercorrelation between the subscales.

### Study 3. Confirmatory factor analysis

A total of 192 women scheduled for hysterectomy completed the SFQ pre-operatively. Median fear response of this sample was higher than the two previous samples with a score of 22.9 (IQR 11–37) on the eight-item version of the SFQ, which is as high as the score on the ten-item version in our initial sample and even higher than the scores of the Portuguese women on the ten-item version (table 2). Similar as in the previous samples the distribution was skewed to the right, and all item scores covered the full range of 0–10.

CFA was performed to compare a one-factor model with the two-factor model as determined by the previous EFA. Table 4 displays the results of the CFA. All test criteria indicated a poor fit of the one-factor model. The two-factor model revealed a fair model fit, except for the RMSEA. Cronbach's alpha was 0.89 for the total scale and 0.86 and 0.87 for the SFQ-s and SFQ-I respectively. Intercorrelation between the SFQ-s and SFQ-l subscale was 0.61, *p*<0.01.

**Table 4:** Confirmatory factor analysis.

	Study 3		Study 4	
	1 Factor	2 Factors	1 Factor	2 Factors
Minimum Fit Function chi square (df)	251.9179 (20)	88.6924 (19)	1212.4356 (20)	346.5056 (19)
RMSEA	0.2730	0.1357	0.2495	0.1206
Standardized RMR	0.1010	0.0586	0.0838	0.0419
NNFI	0.6435	0.8872	0.7356	0.9236
CFI	0.7476	0.9235	0.8112	0.9481

Minimum Fit Function chi square: improvement of 2 factor model compared to 1 factor model 163.2255 (df 1), *p*<0.0001 (Study 3) and 865.93 (df1) *p*<0.0001 (Study 4). Df: degrees of freedom. RMSEA: Root Mean Square Error of Approximation, Standardized RMR: Standardized Root Mean Square Residual, NNFI: Non-Normed Fit Index, CFI: Comparative Fit Index.

Thus, based on factor analyses in the first three studies, the two-factor model seems most appropriate for the SFQ. However, the second EFA and the CFA were both performed in an entirely female sample undergoing hysterectomy in an inpatient setting. To exclude that these results are population specific, we repeated the CFA in male and female patients undergoing various procedures in day surgery setting. It may be expected that these procedures are more minor and possibly elicit less fear. Because the SFQ is meant to be generally applicable in all kind of surgical settings, generalizability of the results to another setting is important.

### Study 4. Confirmatory factor analysis: generalization to day surgery patients

The eight-item SFQ was completed by 1275 patients at home in the week before surgery. In contrast to our expectation, day surgery patients scored equally high on the SFQ as inpatients, with a median score of 22 (IQR 11–36). This was also true for the subscale fear of long-term consequences, see table 2. All item scores covered the full range of 0–10. Similar to the results in the inpatient sample, the one-factor model did not show adequate fit, whereas the parameters of the two-factor model indicated a fair model, except for the RMSEA. Cronbach's alpha was excellent, i.e. 0.91 for the total scale and 0.88 and 0.89 for the SFQ-s and SFQ-I respectively. Intercorrelation between the SFQ-s and SFQ-l subscale was 0.65, *p*<0.01.

In sum, the SFQ can best be conceived as an eight-item questionnaire consisting of two subscales, with four items measuring fear of the short-term consequences of surgery and four items measuring fear of long-term consequences. The factor structure appears to be robust across different populations and in different languages (Dutch vs. Portuguese). In the next step we assessed the convergent validity of the SFQ with other instruments that have been used to measure pre-operative anxiety or worries, i.e. the PCS, the HADS and the STAI. Also we correlated the SFQ score with pre-operatively assessed expected pain after surgery.

### Study 1–5. Convergent validation

Data were obtained from the four studies presented above and from Study 5, which was specifically set-up to further examine convergent validity. Median score on the SFQ in this latter study was 25 (IQR 10–39.3), see table 2. Cronbach's alpha was again excellent with 0.92 for the SFQ, 0.88 for SFQ-s and 0.90 for SFQ-l. Intercorrelation between the SFQ-s and SFQ-l subscale was 0.73, *p*<0.01.

Table 5 shows the Pearson correlation coefficients between the SFQ and its subscales with pain catastrophizing, expected pain, and general anxiety for all five studies. To facilitate comparison of the results across the five studies, correlation coefficients were calculated using the SFQ eight item version. Correlations between the SFQ and the three other scales were significant at 0.01 level. The correlation with pain catastrophizing ranged from 0.32 to 0.60 and with expected pain from 0.33 to 0.48. For the two studies assessing state anxiety (HADS or STAI-state anxiety subscale) correlations with SFQ were 0.56 and 0.70 respectively. In most cases the correlations with the SFQ total score were slightly higher compared to the correlations with the SFQ-s and SFQ-l. In Study 5 also the STAI-trait anxiety subscale was assessed. The correlation between the SFQ and trait anxiety was significant, but the values of 0.45 for the SFQ, 0.40 for the SFQ-s, and 0.42 for the SFQ-l were lower compared to state anxiety.

**Table 5:** Correlations of the SFQ with pain catastrophizing, expected pain, and state anxiety.

			Study 1	Study 2	Study 3	Study 4	Study 5
Pain Catastrophizing	/	SFQ	0.41^1^	0.44^2^	0.32^1^	0.45^1^	0.60^1^
		SFQ-s	0.34	0.36	0.28	0.41	0.47
		SFQ-l	0.40	0.41	0.31	0.42	0.66
Expected Pain	/	SFQ	0.33	-	0.39	0.48	0.45
		SFQ-s	0.33	-	0.42	0.46	0.40
		SFQ-l	0.26	-	0.27	0.42	0.42
State anxiety	/	SFQ	-	0.56^3^	-	-	0.70^4^
		SFQ-s	-	0.53	-	-	0.62
		SFQ-l	-	0.40	-	-	0.66

Pearson correlation, all significant at 0.01 level. - Not applicable.

SFQ: eight items; SFQ-s: Surgical Fear Questionnaire short-time consequences (item 1–4), SFQ-l: SFQ long-term consequences (item 5, 6, 9, and 10).

Catastrophizing: ^1^PCS: Pain Catastrophizing Scale, (Study 1, 3 and 5 13 items; Study 4 six items: I feel I can't stand it any more, I become afraid that the pain may get worse, I can't seem to keep it out of my mind, I keep thinking about how badly I want the pain to stop, there is nothing I can do to reduce the intensity of the pain, I wonder whether something serious may happen). Catastrophizing: ^2^CSQ-c: Coping Strategies Questionnaire-Revised, subscale pain catastrophizing.

State anxiety: ^3^HADS-a: Hospital Anxiety and Depression Scale, anxiety subscale.

State anxiety: ^4^STAI: State-Trait Anxiety Inventory, state subscale.

Thus, the SFQ appeared to be significantly related to other instruments used to assess pre-operative anxiety or worry, in particular to the HADS and the STAI-state anxiety subscale. In the next step we looked at the sensitivity of the SFQ to detect the hypothesized differences in fear in certain subgroups. In accordance with previous studies, we expected that female patients, younger patients, and patients with less education would score higher on surgical fear. The other factors were included exploratory.

### Study 1–5. Sensitivity to differences in patient characteristics

To assess the effect of different patient characteristics on the SFQ the following subgroups were defined: age <65 years compared to ≥65 years (in Study 3 patients older than 65 years were excluded), males compared to females, lower compared to intermediate/higher education, employed compared to not employed, ASA classification I/II compared to ASA III/IV, malignancy as indication for surgery yes/no, and preoperative pain <40 compared to ≥40 on 100 mm VAS. In table 6 the results of the sensitivity analyses are presented, again using the SFQ eight-item score across all five studies. In general, the results for the SFQ-s and SFQ-l subscales were in line with the SFQ total score. As expected, SFQ scores of the younger participants were higher compared to those reported by older participants although only in the Portuguese sample this difference reached statistical significance. Also in line with our expectations, females appeared to be more fearful about the surgery compared to males (Study 1 and 4 significant, Study 5 non significant (ns)). Concerning the effect of education on surgical fear, a difference between the Portuguese and the Dutch populations occurred: in the Portuguese population lower education level was associated with a lower level of surgical fear (ns) whereas in all four Dutch populations lower educated participants scored higher compared to intermediate or higher educated participants. In two out of five studies participants with an occupation scored significantly higher on the SFQ compared to participants without an occupation. ASA-classification did not affect SFQ scores. SFQ results for the subgroups concerning malignancy or not as indication for surgery revealed no differences in the studies 1 and 4 with large population samples. In the smaller Study 5 malignancy did lead to significantly increased surgical fear. Finally, preoperative pain was associated with increased surgical fear across all five studies.

**Table 6:** Sensitivity to differences in patient characteristics.

	Study 1	Study 2	Study 3	Study 4	Study 5
Age					
<65	20 (9–33)	14 (6–25)**	22.9 (11–37)	23 (12–37)	25 (11–40)
≥65	19 (8–33)	6 (0–15)	NA	21 (8–35)	22 (6–35)
Sex					
Male	15 (6–27)***	NA	NA	19 (8–31)***	^a^17 (9–34)
Female	24.5 (12.9–36.7)	13 (5–24)	22.9 (11–37)	26 (13.3–40)	26 (12–44)
Education					
Low	22 (8.8–34.1)	^a^12 (4.3–23.8)	25 (13.5–41)	^b^25 (11.3–39)*	28 (6–44)
Intermediate & high	19 (9–31)	19.5 (11.8–24.5)	21 (10–36)	22 (11–35.7)	25 (10.3–38.8)
Employment					
Occupation	^a^21 (10–34)**	^c^14 (6–28)*	24 (11.3–36)	^b^22 (11–36)	25 (10.8–38.3)
No occupation	18 (8–30)	11.5 (2.8–22)	20 (8–38)	23 (11–38)	22.5 (6.5–39.3)
ASA					
I/II	20 (9–33)	14 (5–24)	24 (11–37)	22 (11–36)	^a^25 (11.3–39.8)
III/IV	20.6 (9.5–33.6)	12 (8–23)	10 (4–NC)	25 (12.5–36)	9.5 (3–25)
Malignancy					
Yes	20 (9–33)	NA	NA	24.5 (13–40)	^a^36 (25–58)*
No	20 (9–33)			22 (11–36)	23 (10–38)
Preoperative pain					
No/mild	19 (8–32)***	14 (6.5–22)	18.5 (7.8–32.3)	18 (9–31)***	17 (6–31)**
Moderate/high	26.1 (13.5–39)	15 (5–25)	26 (14–40)	29 (16–42)	35 (14–43)

SFQ (eight items), median (interquartile range). *p<0.05, **p<0.01, ***p<0.001. NA not applicable: hysterectomy patients only, malignancy excluded; in Study 3 age ≥65 excluded. NC not calculable. ASA: American Society of Anesthesiologists.

Deviation of SFQ-short term and SFQ-long term subscale from the SFQ results is indicated as: ^a^SFQ-s significant difference and SFQ-l non significant difference; ^b^SFQ-s non significant difference and SFQ-l significant difference; ^c^SFQ-s and SFQ-l non significant difference.

Preoperative pain: no/mild or VAS/NRS <40, moderate/high or VAS/NRS 40–100, Study 1: pain at time of completion questionnaire, Study 2–5: average pain last week.

The final part of this paper presents data on the predictive validity of the SFQ for acute and chronic post-operative pain and for perceived recovery. We also compare the predictive value of the total SFQ score with that of its two subscales.

### Study 1 and 4. Predictive validity

Median split was used to identify fearful and non-fearful patients. Using OR's generated by bivariate logistic regression analyses, the predictive value of the SFQ for pain and recovery was assessed. Pain scores were dichotomized using a cut off value of 40 for the VAS/NRS. Values of 80–100% were considered as (near) optimal recovery, on the GSR scale of 0–100%. Results are presented in table 7. Acute pain as well as long-term pain was more strongly predicted by the scores on the SFQ-l subscale than the scores on the SFQ-s subscale. Also for recovery the SFQ-l was the strongest predictor. The predictive value of the SFQ total score was in most cases only slightly lower than that of the SFQ-l score. Predictive value of the two subscales of the SFQ for post-operative pain and perceived recovery using multivariate logistic regression analyses was previously reported in studies of Gramke et al. [36] and Peters et al. [17].

**Table 7:** Predictive validity of the SFQ, SFQ-s and SFQ-l.

		Study 1		Study 4	
Outcome	Predictor	OR (95 CI)		OR (95 CI)	
APSP	SFQ	2.73	(1.59–4.69)***	2.35	(1.81–3.04)***
APSP	SFQ-s	1.83	(1.08–3.10)	2.12	(1.64–2.73)***
APSP	SGQ-l	3.55	(1.99–6.32)***	2.62	(2.02–3.39)***
					
CPSP	SFQ	1.77	(1.25–2.51)**	2.28	(1.56–3.34)***
CPSP	SFQ-s	1.66	(1.16–2.37)**	1.66	(1.15–2.39)**
CPSP	SGQ-l	1.77	(1.24–2.51)**	3.05	(2.06–4.51)***
					
GSR	SFQ	0.44	(0.31–0.62)***	0.56	(0.41–0.77)***
GSR	SFQ-s	0.61	(0.43–0.87)**	0.77	(0.56–1.05)
GSR	SGQ-l	0.44	(0.31–0.63)***	0.40	(0.29–0.55)***

Bivariate logistic regression with median split SFQ, SFQ short term and SFQ long term as predictor. APSP: acute postsurgical pain on day 4. CPSP: chronic postsurgical pain after 6 months in Study 1, after one year in Study 4. Pain scores were dichotomized using a cut of value of 40 for the VAS/NRS. GSR: global surgical recovery on a scale of 0–100%, values of 80–100% were considered as good recovery; long term GSR after 6 months in Study 1, after one year in Study 4.

To adjust for multiple testing a Bonferroni correction was applied: a *p*-value <0.017 was considered statistically significant. **p<0.01, ***p<0.001.

## Discussion

The aim of the present study was to establish the reliability and validity of the SFQ. Therefore use was made of data obtained from 5 prospective studies (N = 3233) in inpatient or day surgery patients. Exploratory and confirmatory factor analyses indicated that a two-factor model best describes the structure of the SFQ. Two four-item subscales can be distinguished: fear of immediate consequences of surgery and fear of the long-term consequences. However, the high internal consistency of the SFQ (eight-item total score) and the moderate, but significant intercorrelations between the SFQ short-term and long-term subscales indicate that the SFQ total score may also be suitable for use in studies on surgical fear. This is further attested by the almost comparable predictive value of the SFQ total score compared to the SFQ-l subscale, and both being stronger related to the patient-reported outcomes than the SFQ-s subscale.

Significant intercorrelations with other validated instruments for the measurement of preoperative fear such as pain catastrophizing, expected postoperative pain and state anxiety indicate good convergent validation of the SFQ. As we expected, the SFQ can be used in day surgery as well as in inpatient surgery and has an adequate sensitivity for differences with regard to sex and age, and in the Dutch samples also for education level.

A limitation of this paper is that the SFQ in all five studies was assessed once in the week or evening before surgery. There are no data yet on the effect of preoperative time course on SFQ scores. For coronary surgery patients, Koivula [5] assessed surgical fear during the waiting period at home, at hospital admission, and after surgery. Preoperative fear and anxiety levels were highest during the waiting period at home and dropped after hospital admission. But for other types of surgery most studies only measure preoperative fear or anxiety in the week before surgery [37]–[41]. However, in the case of undesirable high levels of preoperative fear, treatment will be advocated. Depending on the type of intervention, a certain amount of time may be needed before the intended reduction of surgical fear can be achieved. Therefore, to enable preoperative treatment of surgical fear, as well as to further explore the optimal time point for the assessment of surgical fear, a study measuring the SFQ at different time points, starting from preoperative screening until the day of surgery is necessary. Secondly, the differences in SFQ scores between the Portuguese population and the Dutch population with regard to age, sex, education, and employment status raise the question to what extent sociodemographic factors affect the SFQ. Therefore, the stability of the SFQ across different subgroups needs further exploration. Thirdly, because of the non parametric distribution of the SFQ a median split was used for predictive logistic regression analyses. However, for practical use, e.g. selection of the most fearful patients for preoperative treatment of surgical fear, a more stringent cut-of point seems indicated.

Implications for practice. This paper demonstrated that the SFQ is a concise and generic instrument for the assessment of surgical fear, suitable for most types of elective adult surgery. For further research we suggest additional testing of the convergent validation using biomarkers such as preoperative stress hormone levels. Also the effect of linguistic and cultural influences on the SFQ needs further study. Finally, for diagnostic use optimal cut-of points of the SFQ need to be established. We conclude that the SFQ is a valid and reliable eight-item index of surgical fear, consisting of two subscales: fear of the short-term consequences of surgery and fear of the long-term consequences.

## Supporting Information

Questionnaire S1 **Surgical Fear Questionnaire.**
(DOCX)
