# Correction: Validation of the Surgical Fear Questionnaire in Adult Patients Waiting for Elective Surgery

**DOI:** 10.1371/journal.pone.0162737

**Published:** 2016-09-09

**Authors:** Maurice Theunissen, Madelon L. Peters, Erik G. W. Schouten, Audrey A. A. Fiddelers, Mark G. A. Willemsen, Patrícia R. Pinto, Hans-Fritz Gramke, Marco A. E. Marcus

There are three errors in the “Predictor” column of Table 7. For the last APSP, CPSP, and GSR “Outcome” rows, the correct predictor should be SFQ-1, not SGQ-1. Please view the corrected table below.

**Table 7:** Predictive validity of the SFQ, SFQ-s and SFQ-l.

		Study 1		Study 4	
Outcome	Predictor	OR (95 CI)		OR (95 CI)	
APSP	SFQ	2.73	(1.59–4.69)***	2.35	(1.81–3.04)***
APSP	SFQ-s	1.83	(1.08–3.10)	2.12	(1.64–2.73)***
APSP	SFQ-l	3.55	(1.99–6.32)***	2.62	(2.02–3.39)***
CPSP	SFQ	1.77	(1.25–2.51)**	2.28	(1.56–3.34)***
CPSP	SFQ-s	1.66	(1.16–2.37)**	1.66	(1.15–2.39)**
CPSP	SFQ-l	1.77	(1.24–2.51)**	3.05	(2.06–4.51)***
GSR	SFQ	0.44	(0.31–0.62)***	0.56	(0.41–0.77)***
GSR	SFQ-s	0.61	(0.43–0.87)**	0.77	(0.56–1.05)
GSR	SFQ-l	0.44	(0.31–0.63)***	0.40	(0.29–0.55)***

Bivariate logistic regression with median split SFQ, SFQ short term and SFQ long term as predictor. APSP: acute postsurgical pain on day 4. CPSP: chronic postsurgical pain after 6 months in Study 1, after one year in Study 4. Pain scores were dichotomized using a cut of value of 40 for the VAS/NRS. GSR: global surgical recovery on a scale of 0–100%, values of 80–100% were considered as good recovery; long term GSR after 6 months in Study 1, after one year in Study 4.

To adjust for multiple testing a Bonferroni correction was applied: a *p*-value <0.017 was considered statistically significant.

**p <0.01,

***p <0.001.

## References

[1] Theunissen M, Peters ML, Schouten EGW, Fiddelers AAA, Willemsen MGA, Pinto PR, et al (2014) Validation of the Surgical Fear Questionnaire in Adult Patients Waiting for Elective Surgery. PLoS ONE 9(6): e100225 doi:10.1371/journal.pone.0100225 24960025 10.1371/journal.pone.0100225 PMC4069058

