# Supplementary material for: Validation of the Surgical Fear Questionnaire in Adult Patients Waiting for Elective Surgery
Source: PLoS One. 2014 Jun 24;9(6):e100225. doi: 10.1371/journal.pone.0100225 (PMC4069058; doi:10.1371/journal.pone.0100225)
Supplement: Questionnaire S1 — Surgical Fear Questionnaire. (DOCX) [file pone.0100225.s001.docx]

S1 Surgical Fear Questionnaire

Patient instructions:

This questionnaire assesses how afraid you are for various aspects related to the surgical procedure you are about to undergo. Please circle the number that best reflects how you feel right now.

1. I am afraid of the operation
2. I am afraid of the anaesthesia
3. I am afraid of the pain after the operation
4. I am afraid of the unpleasant side effects (like nausea) after the operation
5. I am afraid my health will deteriorate because of the operation
6. I am afraid the operation will fail
7. (I am afraid of staying in the hospital)*
8. (I worry about my family)*
9. I am afraid that I won’t recover completely from the operation
10. I am afraid of the long duration of the rehabilitation after the operation

| \| 0 \| 1 \| 2 \| 3 \| 4 \| 5 \| 6 \| 7 \| 8 \| 9 \| 10 \| \| --- \| --- \| --- \| --- \| --- \| --- \| --- \| --- \| --- \| --- \| --- \| \| not at all \|  \|  \|  \|  \|  \|  \|  \|  \|  \| very \| \| afraid \|  \|  \|  \|  \|  \|  \|  \|  \|  \| afraid \| | | |
| --- | --- | --- | --- | --- | --- | --- | --- | --- | --- | --- | --- | --- | --- | --- | --- | --- | --- | --- | --- | --- | --- | --- | --- | --- | --- | --- | --- | --- | --- | --- | --- | --- | --- | --- | --- |
|  |  |  |
|  |  |  |

*Note: in the final version item seven and eight are left out.

For the calculation of the total score the following instructions are applicable: one missing item score at maximum is allowed, to be replaced by the subject’s mean score. In the case of more than one missing, the SFQ should not be interpreted. For the calculation of the subscales (short-term fear item 1-4 and long-term fear item 5, 6, 9, 10) no missings are allowed. If a subject enters two scores for one item: if adjacent, choose the highest value, if non-adjacent (other values are in between) the item has to be considered as missing.
